# A shared pattern of midfacial bone modelling in hominids suggests deep evolutionary roots for human facial morphogenesis

**DOI:** 10.1098/rspb.2023.2738

**Published:** 2024-04-17

**Authors:** Alexandra Schuh, Yann Heuzé, Philipp Gunz, Michael A. Berthaume, Colin N. Shaw, Jean-Jacques Hublin, Sarah Freidline

**Affiliations:** ^1^ CNRS, Ministère de la Culture, PACEA, UMR 5199, Université de Bordeaux, Bât. B2, Allée Geoffroy Saint-Hilaire, Pessac 33615, France; ^2^ Department of Human Origins, Max Planck Institute for Evolutionary Anthropology, Deutscher Platz 6, Leipzig 04103, Germany; ^3^ Department of Engineering, Faculty of Natural, Mathematical and Engineering Sciences, King's College London, London, UK; ^4^ Department of Evolutionary Anthropology, University of Zurich, Zurich, Switzerland; ^5^ Chaire de Paléoanthropologie, Collège de France, Paris, France; ^6^ Department of Anthropology, University of Central Florida, Orlando, FL, USA

**Keywords:** facial evolution, ontogeny, bone formation, bone resorption, geometric morphometrics

## Abstract

Midfacial morphology varies between hominoids, in particular between great apes and humans for which the face is small and retracted. The underlying developmental processes for these morphological differences are still largely unknown. Here, we investigate the cellular mechanism of maxillary development (bone modelling, BM), and how potential changes in this process may have shaped facial evolution. We analysed cross-sectional developmental series of gibbons, orangutans, gorillas, chimpanzees and present-day humans (*n* = 183). Individuals were organized into five age groups according to their dental development. To visualize each species's BM pattern and corresponding morphology during ontogeny, maps based on microscopic data were mapped onto species-specific age group average shapes obtained using geometric morphometrics. The amount of bone resorption was quantified and compared between species. Great apes share a highly similar BM pattern, whereas gibbons have a distinctive resorption pattern. This suggests a change in cellular activity on the hominid branch. Humans possess most of the great ape pattern, but bone resorption is high in the canine area from birth on, suggesting a key role of canine reduction in facial evolution. We also observed that humans have high levels of bone resorption during childhood, a feature not shared with other apes.

## 1. Introduction

Hominoids show large variation in facial shape and size [1]. Orangutans possess upwardly oriented faces in relation to the basicranium, a condition called airorhynchy [2]. In contrast, African great apes are klinorhynch, with the midface rotated ventrally and posteriorly [2]. The short and retracted face of humans is distinct from the long, forwardly projecting faces of non-human great apes. Similarly, in gibbons the midface is also less prognathic than in other hominoids [3–5]. The morphology of the dental arcade is U-shaped in apes and parabolic in humans [6]. Additionally, human evolution is marked by smaller canine teeth and less difference in size between males and females [7,8]. However, the underlying ontogenetic mechanisms explaining variation in hominoid facial morphology remain unclear.

A way to study the ontogeny of morphological features, is by looking at the microscopic development of the bone. Bone modelling (BM) is the cellular process of bone growth and development. Along with sutural growth, it is the key mechanism that participates in bone morphogenesis. During the development of skeletal features, the two opposite cellular activities of bone formation and resorption work in concert in order to model and increase the size of the bones. Donald Enlow was the first to propose a link between the cellular activity and the shape of bone [9]. Later, it was noted that bone formation and resorption both leave specific marks on dry bone's periosteal surface, which can be visualized with non-invasive microscopic techniques [10]. Among primates, the human facial BM pattern has been investigated the most [11–17]. The human pattern is described as unique and derived, characterized by high levels of bone resorption in the maxilla in relation to the small human face [18,19]. However, when this change in the cellular pattern occurred in the course of human evolution remains elusive. The study of BM in extant hominoids can thus shed light on major evolutionary events regarding facial morphogenesis.

Studies focusing on other primate species and fossil hominins are scarce. Previous work proposed that patterns of BM are variable, but species-specific. Contrary to humans, bone resorption is less present in gorillas and chimpanzees who display larger amounts of bone formation in the face [19,20]. Previous work on early hominins suggested that *Australopithecus* and *Paranthropus* possess opposite patterns of BM, with *Australopithecus* showing no bone resorption and projecting faces, and *Paranthropus* showing resorption in the premaxilla associated with more vertical faces [21,22]. However, as most previous studies suffered from a lack of quantitative data, there is no consensus about how variable BM is in great apes, which limits our ability to study BM in fossils. Moreover, a shared ancestral pattern in all hominids has also been suggested [23–25], such as similarities in BM between gorillas and chimpanzees [20], as well as between chimpanzee and humans [19]. To verify the hypothesis of a shared pattern, we compare maxillary development in several hominoid species (gibbons, orangutans, gorillas, chimpanzees and humans). We investigate the development of facial prognathism, and aim to identify key ontogenetic events that led to changes in facial morphology. We use an integrative approach that combines techniques of geometric morphometrics (GM) to quantify and visualize the shape changes on the macroscopic scale, as well as surface histology, to quantify and visualize the cellular changes on the microscopic scale.

## 2. Material and methods

### (a) Sample

The number of individuals varied depending on the analysis (BM surface analysis and GM shape analysis). We studies cross-sectional ontogenetic series of gibbons (represented by several species: *Hylobates Iar, H. muelleri, H. agilis* and *H. molloch*; BM: *n* = 24, GM: *n* = 18; Naturkunde Museum, Berlin, Germany), orangutans (*Pongo pygmaeus*; BM: *n* = 31, GM: *n* = 38; Naturkunde Museum, Berlin, Germany; Phyletisches Museum, Jena, Germany; Duckworth laboratory, Cambridge, United Kingdom), gorillas (*Gorilla gorilla*; BM: *n* = 24, GM: *n* = 37; Naturkunde Museum, Berlin, Germany; Phyletisches Museum, Jena, Germany), chimpanzees (*Pan troglodytes*; *n* = 33 in both BM and GM analyses; Tai anatomical collection from the Max Planck Institute for Evolutionary Anthropology, Leipzig, Germany) and present-day humans (BM, GM: *n* = 57 in both BM and GM analyses; Anatomical collection of Strasbourg, France; Anatomical Institute of the University of Leipzig, Germany; Coimbra Anthropological collection, Portugal). Individuals were organized into five age groups (AG) according to maxillary dental development such as in [26]: no teeth erupted (AG 1), deciduous dentition (AG 2), first permanent molar (M1) erupted (AG 3), second permanent molar (M2) erupted (AG 4) and third permanent molar (M3) erupted (AG 5). Individuals with apparent pathologies or taphonomically altered surfaces were not studied.

Imprints of the maxillary surface were generated using a low-viscosity silicone (President Plus Light body, Coltène) directly applied onto the bone following [27]. Positive replicas were then created using a transparent epoxy resin (Araldite 2020, Escil). Computed tomographic (CT) images were used to generate three-dimensional models of each individual. The resolution of the scans ranged between 0.03 and 0.4 mm. Surface models were generated using Avizo (Thermo Fisher Scientific).

### (b) Analyses

#### (i) Geometric morphometrics

Using the software Viewbox 4 (dHAL software; Kifissia), we digitized 249 landmarks and semilandmarks (table 1) on the right maxilla of each individual. Estimation of missing landmarks was carried out with a thin-plate spline (TPS) interpolation [28] using the package Morpho in R [29]. We performed the sliding of all the semilandmarks, minimizing the bending energy, in order to assure geometric homology between all individuals [30]. Finally, to standardize the position, orientation and scaling to a unit centroid size, a generalized Procrustes analysis was performed [31]. We computed principal component analyses (PCAs) of the morphological data in shape space and Procrustes form (size and shape) space [32].

**Table 1.** Landmarks and semilandmarks numbers and definition (total: 249).

landmarks	label	
***fixed landmarks***		
superolateral nasion	sln	
dacryon	d	
zygoorbitale	zyo	
inferolateral rhinion	ilr	
anterior nasal spine	ans	
alveolare (infradentale superius)	ids	
zygomaxillare	zm	
malar root origin	mro	
maxillo-palatine suture	mps	
***curve semilandmarks***		***number—definition***
fronto-maxillary suture	FMS	2—superolateral nasion to dacryon
naso-maxillary suture	NMS	6—superolateral nasion to inferolateral rhinion
inferior orbital margin	IOM	6—dacryon to zygoorbitale
nasal aperture outline	NA	6—inferolateral rhinion to anterior nasal spine
subnasal outline	SO	3—nasal spine to alveolar
zygomatico-maxillary suture	ZMS	5—zygoorbitale to zygomaxillare
maxillary contour	MC	4—zygomaxillare to malar root origin
alveolar outline	AO	8—alveolare to maxillo-palatine suture
***surface semilandmarks***		200—covering the whole surface of the bone

To visualize the patterns of shape changes within one species throughout ontogeny, heat maps were computed to show the shape differences between subsequent age group means [33]. Only the differences between AG 1 and AG 3, and between AG 3 and AG 5, were computed so as to enhance the visualization of the results. As AG 1 is missing in our gibbon and gorilla samples, the shape changes between AG 2 and AG 3 were computed instead. Results are shown using the surface model of the youngest age group (i.e. 1 and 3). Warm colours indicate positive differences between the two meshes, while cold colours indicate negative differences. This can be interpreted as forward and backward displacements from the youngest to the oldest age group, respectively [16,20]. Because of interspecific variation in the amount of shape change, maps were computed at different scales for each species to optimize the visualization of the colours. Only the surface in relation to the template shows informative data, which excludes the teeth.

#### (ii) Surface histology

Surface histology was employed to analyse the BM patterns in each species [16,34]. Pictures of the bone surface were taken with a confocal microscope (Sensofar S neox) using a 5× lens. Only one of the two sides (right or left) was chosen depending on the surface preservation. A grid of 5 × 5 mm was drawn directly on the positive replicas and divided into sub-squares of 2.5 × 2.5 mm in areas of interest (i.e. where both bone formation and resorption can be found). The pictures taken in each 2.5 × 2.5 mm square were then analysed in the software ImageJ [34]. Areas of bone resorption were manually selected on the pictures and transformed into percentages for each square of the grid.

To visualize and compare the BM pattern of each species/individual, digital maps were created in R Studio based on the calculated percentages [16]. A colour code is used to represent the amount of bone resorption. Cold tones indicate high percentages of bone resorption while warm tones indicate low percentages of bone resorption. A percentage of zero indicates bone formation. Bone formation can either be represented by areas of intensively newly added bone, or areas where bone is almost quiescent. As individuals of different ages (and thus, sizes) are represented in our sample, each map was standardized to a given grid size (in this case, 8 × 8 squares; see [16] for a description of the method). This standardization step allows for the comparison of all individuals' bone modelling pattern in our analyses.

To visualize the average changes in BM throughout ontogeny, mean BM maps were calculated at each age group and for each taxon. These were projected directly onto their respective mean forms (i.e. the shape retaining the individual maxillary size measured as centroid size) using Geomagic Studio (3D Systems, Research Triangle Park).

To quantify the changes in the amount of bone resorption throughout ontogeny on the periosteal surface of the maxilla, a total percentage of bone resorption (%BR) was calculated for each individual using the calculated amount of bone resorption corrected by the total area of the bone. As this is not indicative of local changes of bone resorption on the maxilla, the percentage of bone resorption was computed and compared for each of the 51 squares of the mean grid, at each age group and for each species.

A PCA was performed on the BM data (represented as the percentages of bone resorption at each square) to investigate the variation in bone resorption within and between species, and a PERMANOVA (1000 permutations) was conducted on the percentages of bone resorption to test for significant differences between species using a Bonferroni correction.

## 3. Results

### (a) Shape analysis

**Figure 1.**
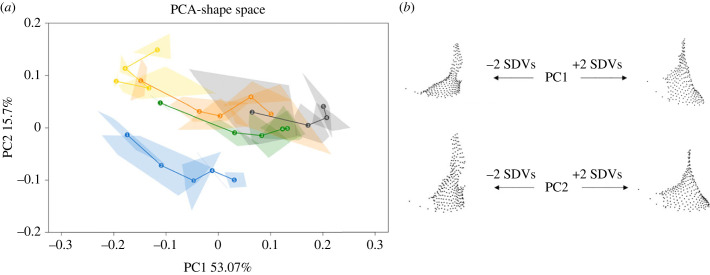
(*a*) Principal component analysis (PCA) in shape space. Developmental trajectories are represented as solid lines linking each age group's mean shape (shown as large dots and numbers). Intra-group variation is represented by convex hulls, and groups with only 2 individuals are linked by a solid line. Blue, humans; green, chimpanzees; orange, orangutans; black, gorillas; yellow, gibbons. (*b*) Landmark configurations representing the extreme shapes at each PC with plus/minus 2 s.d. from the sample mean.

PCA in shape (figure 1*a*) and form space (electronic supplementary material, figure S1) were performed on the maxilla. The PCA in shape space highlighted differences between species in maxillary development. The first PC (PC1) accounts for 53.1% of the total shape variation and PC2 accounts for 15.7%. While the ontogenetic trajectory in all non-human apes occurs in a similar direction in young age groups (from AG 1 to 3), this differs in late stages during which each species’s trajectory diverges (in AG 4 and AG 5). The human trajectory diverges in direction from birth onwards, although it shows a pattern of shape change similar to that of the great apes from AG 1 to AG 3. Results of the permutation test performed on the scores of the first three principal components indicate that humans and gibbons are significantly different from the non-human great apes. Gorillas and orangutans also significantly differ (table 2). The morphological changes associated with PC1 (from negative to positive values) relate to a vertical elongation of the bone, and enlargement as well as a forward development of the premaxilla (figure 1*b*). Changes associated with PC2 (from negative to positive values) relate to an enlargement of the frontal process, as well as the forward development of the premaxilla.

**Table 2.** Permutation test performed on the scores of the first three principal components of the shape data (figure 1). Significant *p*-values are highlighted in italics.

	chimpanzees	gibbons	gorillas	humans
gibbons	*0.01*			
gorillas	0.25	*0.01*		
humans	*0.01*	*0.01*	*0.01*	
orangutans	0.38	*0.01*	*0.01*	*0.01*

**Figure 2.**
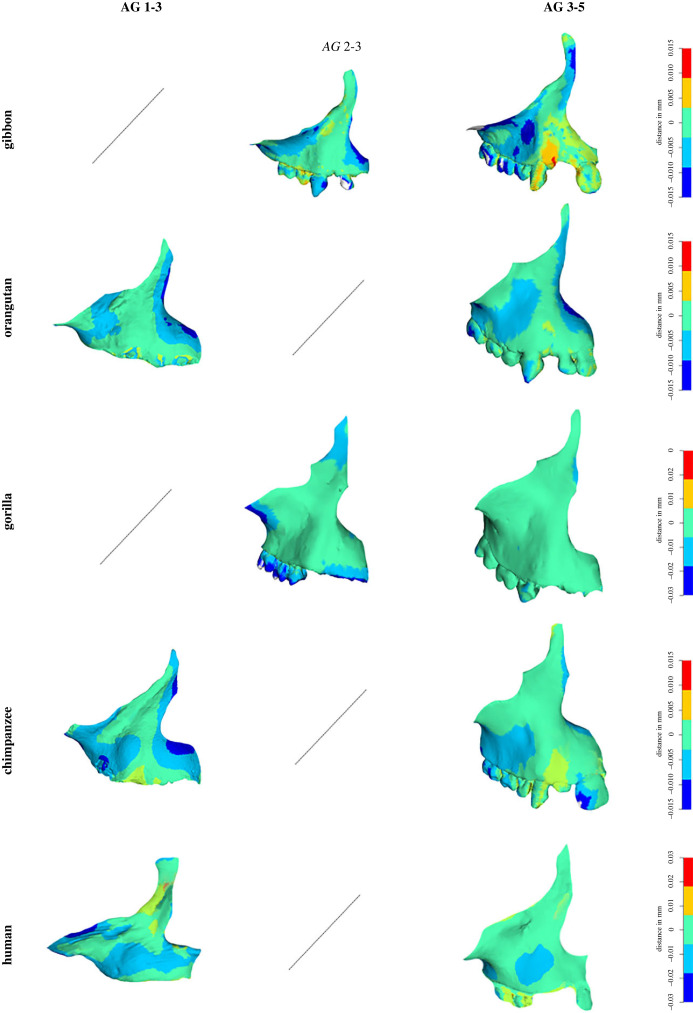
Shape changes between subsequent age groups within species, visualized as heatmaps on the surface of the youngest age group. For better visualization, the changes were computed between AG 1 and AG 3, and between AG 3 and AG 5. As AG 1 is missing in gibbons and gorillas, shape differences were computed between AG 2 and AG 3 instead. The heatmaps were computed with a different colour scale for each species in order to improve the visualization. Only the surface in relation to the template shows informative data, which excludes the teeth.

Figure 2 shows the shape changes between subsequent age groups (AG 1 and AG 3, and AG 3 and AG 5) in each species. Both orangutans and chimpanzees show warm colours in the canine region (indicative of a positive distance, i.e. a forward displacement of this area) while humans show cold colours in this area (indicative of a negative distance, or a backward displacement). In gibbons, such as in orangutans and chimpanzees, the canine region again shows warm colours indicative of a positive difference. Shape changes in the gorilla are more homogeneous across the whole maxilla (i.e. showing green colour indicative of subtle changes), with negative distances found in the frontal process. For all species, the differences between AG 3 and AG 5 were computed. All species, apart from humans and gorillas, show positive values in the canine and canine pillar regions. In gorillas, shape changes are again more homogeneous across the bone, with slightly warmer colours in the premaxilla and zygomatic regions and colder colours in the post-canine region. Similarly, humans show less contrasted colours in the heatmap, with cold colours in the canine area.

### (b) Surface analysis

**Figure 3.**
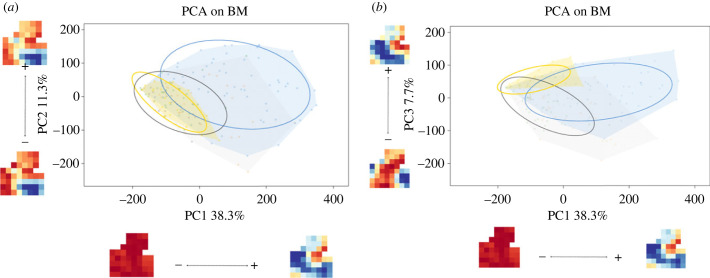
Principal component analysis (PCA) of the bone modelling data. (*a*) PC1 against PC2; (*b*) PC1 against PC3. Humans are shown in blue, the non-human great apes (gorillas, chimpanzees and orangutans) in grey and gibbons in yellow. Ellipses encompass 75% of the total variation.

PCA were performed on the BM data of the whole sample (figure 3; electronic supplementary material, figure S2). The first two PCs represent 49.6% of the total variation (PC1: 38.3%; PC2: 11.3%; figure 3*a*). Although a large overlap is observed (especially between humans and great apes), the majority of the humans plot in the positive values (see 75% ellipse) while the other species plot in the negative values on PC1. They were the only one to show significant different scores with all the other species on PC1 and PC2 (tables 3 and 4). The gibbons all plot in the non-human great ape range of variation. Changes associated with PC1 relate to the increase in bone resorption in the whole bone from the negative to the positive values. Changes associated with PC2 relate to a slight increase in bone resorption in the frontal process and the zygomatico-maxillary suture area from the negative to the positive values.

**Table 3.** Permutation test performed on the scores of the first principal component (PC1) of the bone modelling data (figure 2). Significant *p*-values are highlighted in italics.

	chimpanzees	gibbons	gorillas	humans
gibbons	1.00			
gorillas	1.00	1.00		
humans	*0.01*	*0.01*	*0.01*	
orangutans	1.00	1.00	1.00	*0.01*

**Table 4.** Permutation test performed on the scores of the third principal component (PC2) of the bone modelling data (figure 2). Significant *p*-values are highlighted in italics.

	chimpanzees	gibbons	gorillas	humans
gibbons	1.00			
gorillas	1.00	1.00		
humans	0.38	0.29	*0.01*	
orangutans	1.00	1.00	1.00	*0.01*

The first and third PCs represent 46% of the total variation (figure 3*b*). Gibbons distinguish themselves from the other apes on PC3 (7.7%), with the major axis of the ellipse showing a similar direction to the humans. Table 5 shows the results of the permutation test on PC3 scores. Significant *p*-values are found between gibbons and chimpanzees, gibbons and orangutans, as well as between humans and orangutans. Changes associated with PC3 relate to an increase in bone resorption in the maxillary arcade and frontal process from the negative to the positive values.

**Table 5.** Permutation test performed on the scores of the third principal component (PC3) of the bone modelling data (figure 2). Significant *p*-values are highlighted in italics.

	chimpanzees	gibbons	gorillas	humans
gibbons	*0.01*			
gorillas	1.00	0.17		
humans	0.17	1.00	1.00	
orangutans	1.00	*0.01*	1.00	*0.01*

#### (i) The gibbon bone modelling pattern

**Figure 4.**
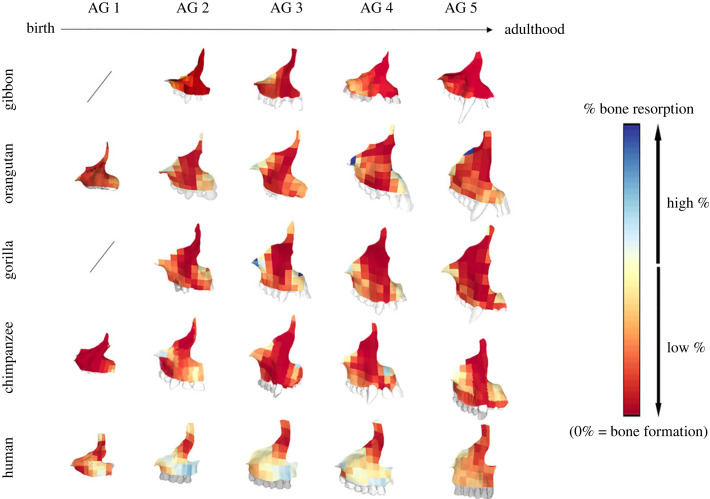
Mean bone modelling maps representing the bone modelling pattern of each age group and species. High percentages of bone resorption are represented in cold tones while low percentages of bone resorption are represented in warm tones. A percentage of 0 indicates bone formation.

We analysed for the first time the maxillary BM pattern of the gibbons (figure 4). The gibbon maxilla shows predominant bone formation in the frontal process and in the nasal area. Bone resorption was only found near the zygomatico-maxillary suture and post-canine regions, with some areas close to the canine. This pattern is repeated from AG 2 to adulthood.

#### (ii) The great ape bone modelling pattern

The computation of the great apes' mean BM maps highlighted different points (figure 4). In all species for which data on individuals at birth were not missing (orangutans, chimpanzees and humans), AG 1 is the ontogenetic stage that diverges the most from the other age groups regarding where bone resorption is found on the bone. Humans already show more bone resorption at birth in the maxillary arcade, with higher percentages of bone resorption located on the canine bulb and near the fronto-maxillary suture. In both of the non-human great apes (orangutans and chimpanzees), resorption is found in the premaxilla.

From AG 2, bone resorption appears in the following areas in all great apes: the premaxilla, the zygomatic process, the top of the frontal process, as well as along the alveolar process and in the post canine area. Humans also possess bone resorption in these regions; however, bone resorption covers the entire maxillary arcade, joining the premaxilla and the post canine regions. This pattern is repeated from AG 2 to AG 5.

#### (iii) Changes in the total amount of bone resorption throughout ontogeny

**Figure 5.**
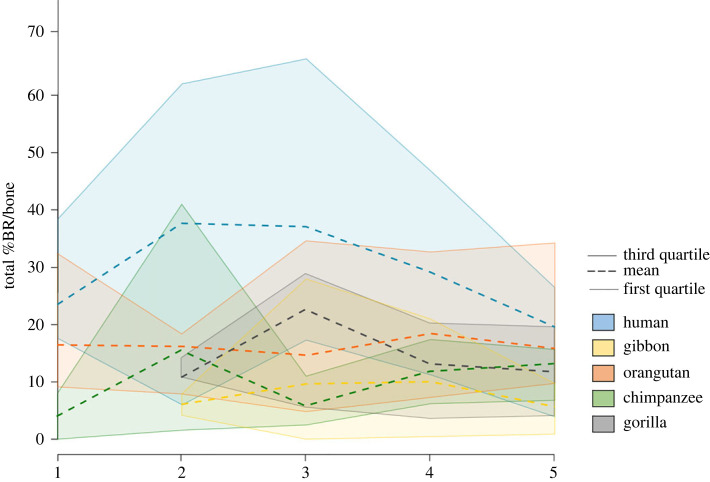
Variation in the total amount of bone resorption in the maxilla throughout ontogeny in all species. Blue, humans; green, chimpanzees; orange, orangutans; black, gorillas; yellow, gibbons. Dashed lines, mean; solid lines, first and third quartiles.

The variation in the total amount of bone resorption for each individual highlighted species’s specific differences in how bone resorption is expressed throughout ontogeny (figure 5). In comparison to the great apes, gibbons show lower amounts of bone resorption throughout life (between 0 and 25%), with a small increase between AG 3 and AG 4. The non-human great apes show a larger range of variation (between 0 and 40%). Each species shows a different pattern, with chimpanzees expressing a peak in bone resorption in AG 2, while gorillas express a peak in AG 3. Orangutans show the most homogeneous pattern throughout life among non-human primates.

Humans express high amounts of bone resorption already from birth, with particularly high amounts expressed in AG 2 and AG 3. A progressive decrease is observed between AG 3 and AG 4, and between AG 4 and AG 5. This pattern is unique to that species.

#### (iv) Local differences in bone resorption

**Figure 6.**
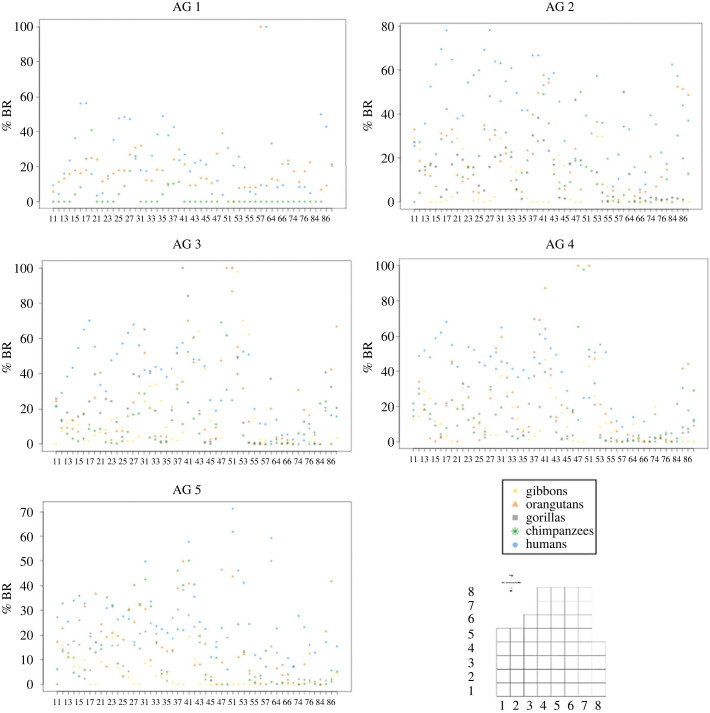
Plots representing the percentages of bone resorption at each square of the grid. *x*-axis, square number; *y*-axis, percentage of bone resorption. Blue dots, humans; green stars, chimpanzees; grey squares, gorillas; orange triangles, orangutans; yellow diamonds, gibbons. A grid is represented to show the location of each square.

To quantify the local variation of bone resorption, we plotted the percentage of bone resorption in each square of the mean grid at each age group (figure 6). When considered locally, bone resorption can be expressed in either high or low amounts, depending on the area, age group and species considered (i.e. the pattern of expression of bone resorption changes with time and between species). For example, in AG 2, the square 86 expresses high %BR in humans and orangutans (respectively, 44% and 51.5%), while low percentages in chimpanzees (19.9%) and gorillas (1.1%). Resorption is absent from this square in gibbons. This pattern changes in AG 3, in which bone resorption is at 0% in chimpanzees and gibbons, 32.5% in gorillas, 42.5% in orangutans and 16.42% in humans.

Moreover, this analysis highlighted that some squares possess consistently higher values in humans than in the other apes. When plotting these squares on a grid, we observed that a large majority of them is located in the canine area (electronic supplementary material, figure S3).

## 4. Discussion

This study investigated for the first time the patterns of maxillary BM of almost all extant apes, and sheds new light on the underlying cellular mechanisms that have acted on the evolution of facial morphology.

### (a) A change in the cellular pattern on the hominid lineage

In this study, we identify areas of bone resorption in great apes that were not previously described in BM studies of Afro-Asian monkeys [35,36], such as the presence of bone resorption in the premaxilla (figure 4). It has been shown that a first step in the evolution of the hominoid face lies in the reduction of facial prognathism [37–39], and that compared to Afro-Asian monkeys, great apes possess a shorter facial length [40]. Our results suggest that this change in facial morphology could relate to a change in the cellular pattern on at least two different levels: firstly, the appearance of new resorptive areas such as discussed above; and secondly, the increase in the amount of bone resorption on the hominid lineage (figures 4 and 5), a process that was previously only attributed to *Homo sapiens* due to the large resorptive area found in this species [23]. To test for a correlation between changes in facial size and BM between the gibbons and the non-human great apes, a multiple multivariate regression was performed between the percentages of bone resorption and the logarithm of the centroid size of each individual. We found a significant but weak correlation between size and BM, indicating that size only played a partial role in the appearance of new resorbing areas in non-human great apes (electronic supplementary material, table S1).

Surprisingly, we found that the gibbon pattern differs the most from our hominoid sample, with areas of bone resorption located mostly in the post canine region (figure 4). We would expect to find more bone resorption in their maxilla, since it is often proposed that prognathism is reduced in gibbons [3]. In this respect, the gibbon maxillary BM pattern is more similar to what is currently known for Afro-Asian monkeys who show bone resorption near the zygomatico-maxillary suture [35,36,41]. Midfacial ontogeny in gibbons may thus imply other mechanisms not quantifiable in this study, such as a differential sutural growth that would play a role in their less prognathic face.

### (b) A shared bone modelling pattern between non-human great apes

This study identified that most areas of bone resorption are shared between great apes (figure 4), although shape differences exist between them from birth on (electronic supplementary material, figure S4), as well as differences in facial orientations, projection and diets [24,42]. Thus, the patterns of BM that were observed at the surface of the bone do not seem related to facial orientation, as orangutans possess similar patterns to chimpanzees and gorillas. Rather, sutural growth might be the main process involved in determining facial prognathism [25,43]. Moreover, the high similarities that we found in the location of bone resorption suggest a major influence of genetic factors in determining the cellular pattern of bone growth, as the cellular development appears constrained despite maxillary shape differences. Based on these results, it is likely that early hominins share this ‘ape-like’ pattern, although differences in BM between *Australopithecus* and *Paranthropus* have been discussed in previous studies [21,22]. *Paranthropus* is less prognathic than *Australopithecus*, and similar to the great apes, shows areas of bone resorption in the premaxilla that have not been found in *Australopithecus*. Considering the strong preservation of the location of bone resorption highlighted by this study, it is likely that *Australopithecus* also express bone resorption in this area. However, it could be expressed on a lesser degree, such as suggested by a previous study [44].

Although the cellular pattern is similar in hominids, shape differences exist. We found that although bone resorption is present in similar areas, its expression varies depending on the species and age group considered (figures 5 and 6). This differential expression of bone resorption throughout ontogeny suggests that differences in timings and rates of the cellular activities are the main cause for morphological variation. Differences in cellular proliferation rates could also explain the differences in morphology observed in our sample, as discussed in other species [45]. On the genetic level, this indicates a differential regulation of genes throughout ontogeny, along with a shared pattern in all species. It was found that while most genes are similar between humans and chimpanzees, the genetic expression (i.e. regulatory patterns) of stem cells differentiated into osteogenic cells differ between the two species [46].

### (c) Human specificities in midfacial development

In humans, facial size is reduced and the maxilla is retracted, showing high percentages of bone resorption from birth on. When looking at the changes in the amount of bone resorption through time in the entire maxilla (figure 5), humans distinguish themselves from the other apes by maintaining high amounts of bone resorption during childhood (AG 2 and AG 3), while it is more constant in all other species. Whether this change is due to a higher number of osteoclast precursors and/or osteoclasts (i.e. differences in cellular differentiation and/or division), or more active resorbing cells, is still unknown. This denotes a change in the cellular expression within the human lineage, possibly already occurring in early phases of embryonic development as studies have suggested that a reduction in the number of neural crest cells reduces the size of the jaws [47–49]. The results of this study highlight parts of the mechanisms behind facial gracilization in present-day humans: high amounts of bone resorption that are maintained throughout childhood, followed by a progressive decrease in the cellular activity towards adolescence, which corresponds to a truncated growth (electronic supplementary material, figure S5). This could be related to a change in the hormonal expression pattern, as such pattern (a reduced activity towards adolescence) has also been observed in the expression of the thyroid-stimulated hormones [50–52].

Finally, a noticeable difference between humans and great apes is found in the canine area, in which humans possess uniquely high percentages of bone resorption (figure 6). The analysis of the pattern of shape change (figure 2) showed that canine development is the main difference maintained throughout ontogeny between humans and the other apes. From birth on, resorption is found on the canine bulb in humans, while it is present in the premaxilla in great apes (figure 4). This early stage difference in the human BM pattern in comparison to the non-human great apes strongly supports the hypothesis of a selection towards the reduction of the canine on the hominin lineage [7].

## 5. Conclusion

This study highlighted key periods during hominid facial evolution and ontogeny. First, we found a change in cellular activity in the hominid lineage. Resorption increases slightly and appears in new areas, such as in the premaxilla. This denotes a fundamental change in the cellular pattern of the hominid maxillary development, that has been described as less prognathic than Afro-Asian monkeys. Secondly, this pattern is similar between hominids, as bone resorption is found in similar areas. This denotes strong developmental canalization of the hominid midface. However, this pattern is differently expressed, suggesting differences in cellular differentiation and proliferation, as well as in genetic regulation. Finally, we found two unique features in humans. One is the high percentage of bone resorption that is maintained during a longer period (corresponding to childhood—AG 2 and AG 3—in our sample) that suggests an extended period of osteoclastic activity. The other is that resorption is uniquely high in the canine, and present from birth on. Thus, selection for canine reduction represented a major step for midfacial evolution in the human lineage. Our study highlights the importance of bone resorption in shaping the facial skeleton and how selection of this process acted to generate new morphologies. Together, these results help to better understand the underlying mechanisms for morphological variability, and will represent a solid framework for future BM studies of fossil remains.

## Data Availability

Data and code are available from Dryad: https://doi.org/10.5061/dryad.000000094 [53].
